# Using willingness-to-pay to establish patient preferences for cancer testing in primary care

**DOI:** 10.1186/s12911-016-0345-9

**Published:** 2016-08-09

**Authors:** Sandra Hollinghurst, Jonathan Banks, Lin Bigwood, Fiona M. Walter, Willie Hamilton, Tim J. Peters

**Affiliations:** 1 School of Social and Community Medicine, University of Bristol, Bristol, UK; 2 Department of Public Health and Primary Care, University of Cambridge, Cambridge, UK; 3 University of Exeter Medical School, Exeter, UK; 4 School of Clinical Sciences, University of Bristol, Bristol, UK

**Keywords:** Willingness-to-pay, Cancer, Diagnostic tests, Primary health care

## Abstract

**Background:**

Shared decision making is a stated aim of several healthcare systems. In the area of cancer, patients’ views have informed policy on screening and treatment but there is little information about their views on diagnostic testing in relation to symptom severity.

**Methods:**

We used the technique of willingness-to-pay to determine public preferences around diagnostic testing for colorectal, lung, and pancreatic cancer in primary care in the UK. Participants were approached in general practice waiting rooms and asked to complete a two-stage electronic survey that described symptoms of cancer, the likelihood that the symptoms indicate cancer, and information about the appropriate diagnostic test. Part 1 asked for a binary response (yes/no) as to whether they would choose to have a test if it were offered. Part 2 elicited willingness-to-pay values of the tests using a payment scale followed by a bidding exercise, with the aim that these values would provide a strength of preference not detectable using the binary approach.

**Results:**

A large majority of participants chose to be tested for all cancers, with only colonoscopy (colorectal cancer) demonstrating a risk gradient. In the willingness-to-pay exercise participants placed a lower value on an X-ray (lung cancer) than the tests for colorectal or pancreatic cancer and X-ray was the only test where risk was clearly related to the willingness-to-pay value.

**Conclusion:**

Willingness-to-pay values did not enhance the binary responses in the way intended; participants appeared to be motivated differently when responding to the two parts of the questionnaire. More work is needed to understand how participants perceive risk in this context and how they respond to questions about willingness-to-pay. Qualitative methods could provide useful insights.

## Background

Shared decision making is a stated aim of several healthcare systems [1, 2]. Involving patients in critical decisions about their care is regarded as not only ethically correct but also as a way of improving quality and “avoiding unwanted and costly medical interventions” [3]. In the UK the philosophy of “no decision about me, without me” has been promoted by the Department of Health [4] and has been applied to many aspects of patient care. However, fully shared decisions can only be made if the asymmetry of information between clinicians and patients is more balanced. Recently, the current imbalance has begun to shift, encouraged by a greater will on the part of clinicians and an increase in readily available information accessible to patients, both from the National Health Service (NHS) and elsewhere.

One notable area where shared decision making has been adopted actively is cancer. Research on patients’ views has informed policy on screening [5] and treatment [6] in accordance with referral guidelines developed by the National Institute for Health and Care Excellence (NICE) [7]. Such information is important, as care plans that incorporate patients’ preferences are more likely to be successful in terms of acceptability and may lead to more efficient use of resources. However, one gap in the evidence is information about patients’ views on testing for cancer with respect to risk: how serious do symptoms have to be, in terms of indicating cancer, for patients to consider a particular test to be worthwhile? The lifetime prevalence of cancer in the UK is more than 30 % [8] and despite falling death rates the fear of cancer is known to be high among the general population [9]. Early diagnosis may improve survival [10] but many early symptoms of cancer, for example, cough, diarrhoea, and headache, far more often indicate a benign condition. General practitioners are faced with the challenge of deciding which patients with such symptoms to refer for diagnostic testing, relying largely on their expertise and limited national guidelines [7]. The risk of failing to investigate a potentially serious symptom has to be weighed against the need to avoid unnecessary anxiety, inconvenience, side-effects and cost from inappropriate investigation.

The study described here is part of a larger study reported more fully elsewhere [11], which used a survey of primary care attenders to investigate preferences for cancer investigation. Three contrasting cancers were chosen as exemplars - colorectal, lung and pancreas – because of their variation in symptoms, type and accessibility of test, treatment, and prognosis. Here, we describe a willingness-to-pay component of the survey, which was designed to enhance the results of the main survey: if the same number of participants opted to be investigated for a particular cancer irrespective of risk level, could the values offered in the willingness-to-pay exercise be used to refine these responses and identify a threshold risk level below which testing was not regarded as worthwhile?

Willingness-to-pay has been used extensively to obtain patient and public valuations for a variety of goods and services in many diverse settings [12]. Despite considerable methodological research into the use of different willingness-to-pay techniques [13] no consensus has emerged as to best practice and it is likely that different methods suit different situations and patient groups [14, 15]. The simplest form of value elicitation is to use an ‘open-ended’ approach whereby the respondent is asked to provide a valuation without any prompting or context; more sophisticated, is a ‘payment scale’ approach where a list of feasible values is offered and the respondent chooses from the list. A bidding approach, which is more refined, has generally come to be preferred to both of these [16, 17]. This method requires the respondent to accept or reject a starting bid (value), which is increased or decreased according to the response and the process continues until a final value is determined. Although this is often a preferred method there is evidence suggesting that responses in a bidding approach tend towards the point at which the bidding starts (starting point bias) [18–20]. This can lead to biased results.

The aim of this study was to develop and administer a willingness-to-pay questionnaire that could be used to elicit the relative values that patients place on diagnostic testing for lung, pancreatic and colorectal cancers. We aimed to identify a risk threshold for each cancer that would indicate when patients choose to be tested in preference to watchful waiting.

## Methods

### Study design

We developed a vignette-based survey with a willingness-to-pay component to determine the likelihood that patients would choose to be tested for colorectal, lung, and pancreatic cancer, using various levels of risk. The key question of what proportion of the population would choose to be tested at each risk level for each cancer was addressed using a simple 'yes/no' alternative. Those responding ‘yes’ to a test proceeded to a willingness-to-pay exercise with the aim of identifying a strength of preference around the binary choice. Cookson suggests that willingness-to-pay exercises that aim to elicit true, absolute values are unreliable because of “budget constraint bias”, where the value given is inflated because of the close focus placed on a particular service [21]; comparative willingness-to-pay may be a way of avoiding this. This study adopted the latter technique, with the aim of identifying relative values to differentiate between ‘yes’ responses by risk level. The survey was designed specifically for this study and was administered using an electronic touch screen tablet computer (an iPad). The iPad application software was custom built, which gave us considerable scope and flexibility in the design. We obtained ethics approval from the South West (Southmead) National Research Ethics Service committee (ref 11/SW/0055). Participants provided oral informed consent.

### Survey design

The survey contained three components. The first section asked for information about participant characteristics, including age, sex, income, education, employment status, ethnicity, experience of cancer (self and family member or close friend), and convenience of the nearest main hospital. Screen shots showing details of the way these questions were asked are included in the Appendix (Fig. 3).

Secondly, we used vignettes to ascertain participants’ attitudes towards testing for cancer. We developed twelve separate vignettes, one for each combination of the three cancers (colorectal, lung, and pancreas) and four different risk levels (1 %, 2 %, 5 %, 10 %). The content of the vignettes was informed by current guidelines and clinical experts on the team. We also undertook qualitative interviews with patients referred for symptoms suspicious of cancer [22], and these were used to validate the vignettes’ depiction of the three diagnostic pathways as experienced by patients. Each vignette contained a description of symptoms, the chance that these might indicate cancer – presented as a percentage, ratio, and pictorially – information about the diagnostic test that would be used, likely treatment, and an indication of the prognosis. The information provided in the vignettes is summarised in Table 1. For each participant, one of the twelve vignettes was generated randomly, thus avoiding any ordering effect, and the respondent was asked to imagine they were in the situation described in the scenario. They were then asked whether they would choose to have the diagnostic test if it were offered.

**Table 1:** Summary of content of 12 vignettes: one for each combination of cancer and risk level

	Colorectal	Lung	Pancreas
symptoms for 1 % risk	Diarrhoea on most days	Coughing on most days…unusually tired	Some stomach pain on most days…lost a few pounds (~1.5-3 kg) in weight
symptoms for 2 % risk	Diarrhoea and stomach pain on most days	Coughing on most days…a little out of breath walking up hills…lost a few pounds (~1.5-3 kg) in weight	Some stomach pain on most days…lost half a stone (3.2 kg) in weight
symptoms for 5 % risk	Unusually tired…blood test shows anaemia	Coughing on most days…coughed blood once	Continuous stomach pain…lost half a stone (3.2 kg) in weight
symptoms for 10 % risk	Intermittent bleeding from the back passage (rectal bleeding)…blood test shows anaemia	Coughing on most days…coughed blood a few times…lost half a stone (3.2 kg) in weight	Continuous stomach pain…lost 1 stone (6.4 kg) in weight
test/investigation	Colonoscopy	Chest x-ray	Ultrasound scan followed by CT scan
treatment	Surgery and chemotherapy	“Difficult to treat”	“Difficult to treat”
prognosis/outlook	Early diagnosis may improve outcome	Early diagnosis may improve outcome	Early detection does not necessarily improve survival

**Fig. 1:**
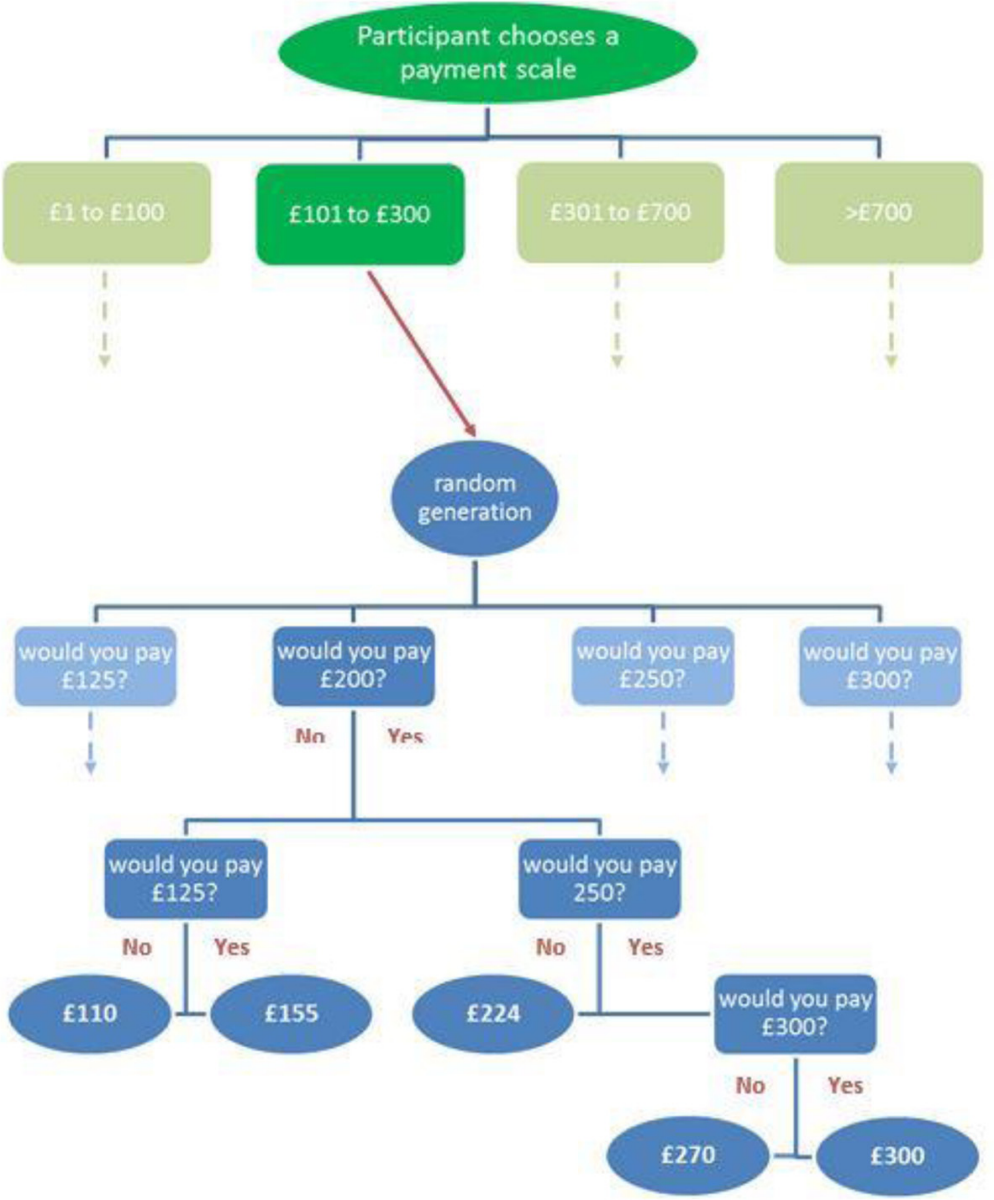
Schema illustrating the two-part willingness to pay exercise. Choice of payment scale leads to a bidding process with the starting point randomly generated from four values within the band

Following a ‘yes’ response to the question about testing, participants proceeded automatically to the third section of the survey – the willingness-to-pay exercise. The design of this part of the survey was informed by experts in the team and with reference to published costs of tests. The survey and the values included were tested on a sample of participants using the technique of verbal probing [23] to check that respondents interpreted the question correctly, and understood why it was being asked. Two rounds of verbal probing were carried out, the first on 13 participants and the second on five. Further pilot testing ensured that the exercise would not be too burdensome. Feedback from the verbal probing was used to refine the content: data from the first round indicated that respondents did not fully understand the concept of opportunity cost so the wording was changed to convey the idea of sacrifice. Subsequent testing in round 2 showed this change was successful. To mitigate starting point (or anchoring) bias we designed a two-part exercise in which respondents were first presented with a payment scale and the response to that question established the starting point of a bidding process. The starting point was generated randomly from a selection within the range of the scale chosen, and the participants could then bid up or down from the starting point. This mechanism is illustrated in Fig. 1, using an example where a participant selects the payment scale £101 to £300. The starting point for the bidding is randomly selected by the software from £125, £200, £250 and £300; in this example £200 is selected. The participant is then able to bid up as far as £300 or down to below £125, with five possible end points. In total, 18 end points were used, five for each of the lower three bands (£1 to £100, £101 to £300, and £301 to £700) and three for the “more than £700” band. Within each band, the difference between each end point and the one immediately higher increased as the value increased so that proportional differences were roughly similar [24].

**Fig. 2:**
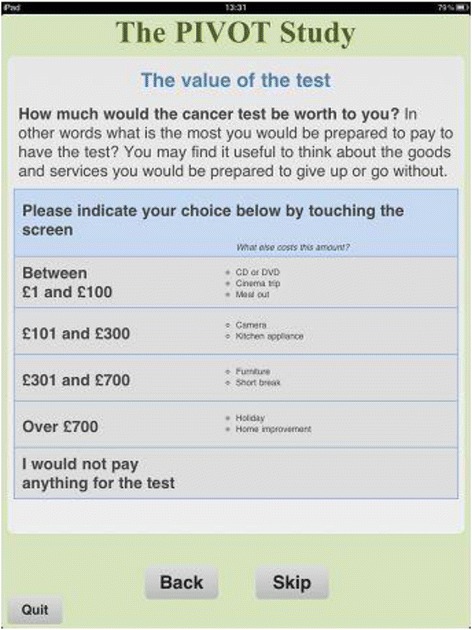
Screen shot of the payment scale exercise showing the use of reference goods

**Fig. 3:**
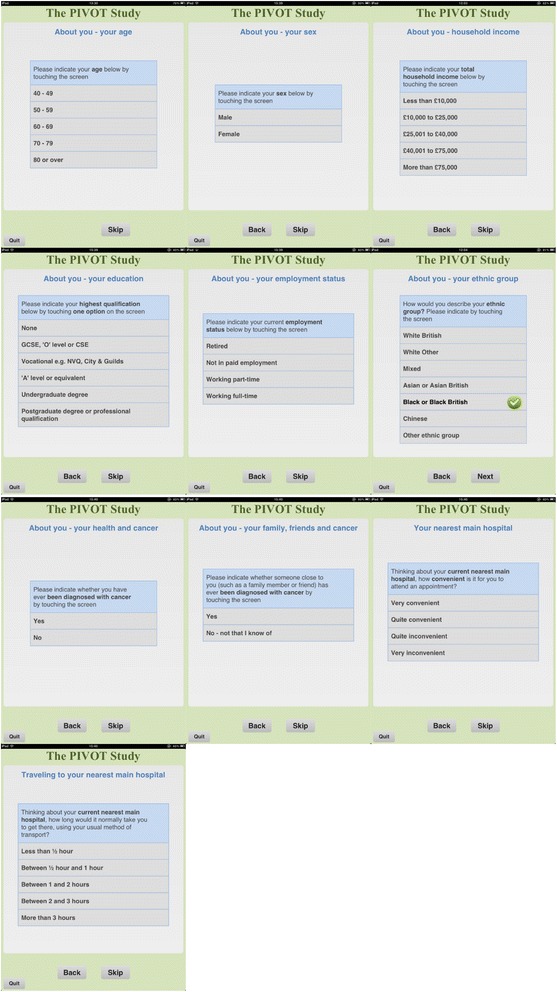
Screen shots showing questions about participant characteristics

At the beginning of the task, when presenting the payment scales, we used reference goods to help participants think about the value of a diagnostic cancer test (see Fig. 2). We chose a selection of ‘lifestyle’ goods and services, seen as being ‘desirable’ though not essential, and which could conceivably be sacrificed to pay for healthcare. Wording was carefully chosen to encourage them to think in terms of sacrifice – that is, what they might be prepared to give up or go without in order to have a test [25].

It is known that some individuals find it hard to place a value on healthcare, particularly in the context of a system of universal coverage as in the UK, and moreover some individuals feel it is unethical to expect them to provide a valuation. To accommodate these views the payment scale offered an option of “I would not pay anything for the test”. If this was selected they were then asked a further question about their reason for this view with the choices of “I cannot afford anything extra”, “I do not believe I should pay for healthcare”, and “It is too difficult to put a value on health”.

Throughout the development of the survey we piloted the wording and layout of all components with a patient and public involvement group using the technique of verbal probing [23]. Feedback from these sessions was used to refine and improve the content.

### Survey administration

The survey was administered by researchers in general practice waiting areas. We targeted a susceptible population – that is, those at greater risk of cancer who would be likely to seek health care help and advice (GP attenders aged 40 and over). General practices in three geographical areas (Bristol & South Gloucestershire, Devon, and the East of England) were included and practices were purposively sampled to achieve an overall mix of urban and rural, and a range of socio-economic statuses. Data collection took place at 26 practices at different times of the day and week between December 2011 and August 2012.

Participants could complete up to three vignettes, one for each cancer. The first vignette was randomly generated from all 12 possibilities (three cancers, four risk levels), the second from the eight that related to the two remaining cancers, and the third from the four relating to the final cancer.

When data collection was complete we conducted a test-retest exercise in a different practice using a convenience sample of 48 volunteers who agreed to return two weeks later. The random generation of vignettes was removed from the survey for the second stage of this exercise to ensure the two tests were identical.

### Analysis

Data were electronically downloaded directly from the iPads at the end of each session. Participant characteristics were explored descriptively and the age/sex profile was compared with that of England as a whole and general practice attenders. Responses to the choice of whether to be tested were analysed descriptively and using logistic regression to establish the extent to which risk played a part in participants’ decisions.

The data from the willingness-to-pay exercise were analysed to explore whether they could be used to inform the strength of preference about the simple 'yes/no' choice. We therefore investigated the extent to which willingness-to-pay values differed according to risk. We explored this descriptively using means and medians, and tested the relationship between the willingness-to-pay values and risk using regression analysis. A one-way analysis of variance, with three degrees of freedom, was conducted to investigate the difference in mean willingness-to-pay across all risk levels, for each cancer.

Analysis was carried out using Stata v13.1 statistical software and Microsoft Office Excel 2013.

## Results

### Participant characteristics

A total of 3,469 participants took part, completing 6,930 vignettes. The characteristics of the participants are shown in Table 2. The age/sex profile of responders is similar to that of the consulting population in England [26]. As expected, when compared with the general population of England the sample was under-represented in terms of younger men (17 % vs 27 % aged 40–59), and over-represented in respect of older women (15 % vs 11 % aged 60–69), and for all elderly people (29 % vs 24 % aged 70 and over) [27]. The respondents were largely white British and nearly half were retired; 15 % had previously been diagnosed with cancer and 75 % had a family member or close friend who had experienced cancer.

**Table 2:** Participant characteristics

Characteristic	Category	*n*	%
*Age group n* = 3452	40-59	1519	44.0
	60-69	945	27.4
	70+	988	28.6
*Sex n* = 3461	male	1457	42.1
	female	2004	57.9
*Income n* = 2958	<£10,000	720	24.3
	£10,000 - £25,000	1166	39.4
	£25,001 - £40,000	581	19.6
	£40,001 - £75,000	319	10.8
	>£75,000	172	5.8
*Ethnicity n* = 3453	White British	3,096	89.7
	White Other	159	4.6
	Mixed	40	1.2
	Asian or Asian British	90	2.6
	Black or Black British	46	1.3
	Chinese	10	0.3
	Other Ethnic Group	12	0.4
*Education n* = 3388	None	1,001	29.6
	GCSE or equivalent	781	23.1
	Vocational / ‘A’ level	850	25.1
	Degree and higher	756	22.3
*Employment n* = 3446	Retired	1,673	48.5
	Not in paid employment	379	11.0
	Working part time	607	17.6
	Working full time	787	22.8
*Cancer diagnosis – self n* = 3463	Yes	522	15.1
	No	2941	84.9
*Cancer – family/ close friend n* = 3465	Yes	2597	75.0
	No	868	25.1
*Convenience of hospital n* = 3461	Very convenient	1,388	40.1
	Quite convenient	1,621	46.8
	Quite inconvenient	323	9.3
	Very inconvenient	129	3.7
*Travel time to hospital n* = 3463	<0.5 h	1,759	50.8
	0.5 – 1 h	1,458	42.1
	>1 h	246	7.1

### Binary responses

Detailed results of the responses to the 'yes/no' question about whether to opt for a test have been reported elsewhere [11]. Table 3 shows the number of participants choosing to be investigated at each risk level for each cancer. A large majority (88 %) of participants chose to be referred for a test; this was slightly lower in the low risk (1 %) group and higher in the high risk (10 %) group, but the difference was very small (87 % vs 89 %). Colonoscopy (colorectal cancer) had a lower uptake than chest X-ray (lung cancer) and ultrasound/CT scan (pancreatic cancer) and displayed the greatest risk gradient. These observations were confirmed by the results of the logistic regression analysis, which controlled for patient characteristics.

**Table 3:** Number (%) choosing to be investigated by cancer and risk level

	Colorectal		Lung		Pancreas		All three cancers (first vignette only)	
Risk level	Number of responses	Number (%) choosing to be tested	Number of responses	Number (%) choosing to be tested	Number of responses	Number (%) choosing to be tested	Number of responses	Number (%) choosing to be tested
1 %	572	462 (81 %)	581	533 (92 %)	582	525 (90 %)	898	782 (87 %)
2 %	569	485 (85 %)	571	531 (93 %)	580	527 (91 %)	838	738 (88 %)
5 %	580	496 (86 %)	589	543 (92 %)	572	526 (92 %)	873	764 (88 %)
10 %	570	508 (89 %)	582	537 (92 %)	582	529 (91 %)	860	768 (89 %)
	2291	1951 (85 %)	2323	2144 (92 %)	2316	2107 (91 %)	3469	3052 (88 %)

### Willingness-to-pay

The results of the willingness-to-pay exercise are presented in Tables 4, 5 and 6. Table 4 gives the number and percentage of participants who selected each payment scale, by cancer and by risk level and Table 5 gives the values indicated in the bidding exercise. Results for each cancer separately are based on all responses, but because participants could respond to up to three vignettes, results for all cancers together use each participant’s first response so as to reduce differential selection bias. Responses covered the entire payment range offered for all cancers and all risk levels, though 68 % of participants bid up to the highest value within the range of the payment scale chosen.

**Table 4:** Number and percentage of respondents selecting each willingness-to-pay band by cancer (all responses) and by risk level (first response only)

	Colorectal	Lung	Pancreas	All three cancers (first response only)
£1-£100	630 (35 %)	774 (39 %)	691 (36 %)	1,030 (37 %)
£101-£300	382 (21 %)	400 (20 %)	397 (20 %)	541 (20 %)
£301-£700	186 (10 %)	180 (9 %)	197 (10 %)	262 (9 %)
over £700	287 (16 %)	278 (14 %)	312 (16 %)	405 (15 %)
would not pay	311 (17 %)	346 (17 %)	340 (18 %)	527 (19 %)
	1,796	1,978	1,937	2,765
	1 %	2 %	5 %	10 %
£1-£100	295 (42 %)	244 (36 %)	242 (36 %)	252 (36 %)
£101-£300	139 (20 %)	127 (19 %)	129 (19 %)	146 (21 %)
£301-£700	58 (8 %)	71 (10 %)	62 (9 %)	71 (10 %)
over £700	83 (12 %)	102 (15 %)	102 (15 %)	118 (17 %)
would not pay	124 (18 %)	137 (20 %)	146 (21 %)	120 (17 %)
	699	681	681	707

**Table 5:** Mean (SD) and median (IQR) willingness-to-pay values, by cancer and risk level

	*n*	Mean (SD) £	Median (IQR) £
Colorectal			
1 %	354	380 (348)	300 (100 to 700)
2 %	351	393 (351)	300 (100 to 700)
5 %	373	367 (351)	270 (100 to 700)
10 %	385	367 (343)	270 (100 to 700)
	1463	377 (348)	300 (100 to 700)
*p*-value		0.71	
Lung			
1 %	401	305 (326)	100 (100 to 300)
2 %	403	339 (336)	270 (100 to 503)
5 %	399	360 (349)	270 (100 to 700)
10 %	412	365 (346)	270 (100 to 700)
	1615	342 (340)	224 (100 to 670)
*p*-value		0.049	
Pancreas			
1 %	389	371 (355)	224 (100 to 700)
2 %	405	367 (336)	300 (100 to 700)
5 %	378	375 (352)	270 (100 to 700)
10 %	401	385 (358)	300 (100 to 700)
	1573	374 (350)	270 (100 to 700)
*p*-value		0.91	
all cancers (first response only)			
1 %	567	312 (327)	100 (100 to 300)
2 %	534	366 (347)	270 (100 to 700)
5 %	529	360 (347)	270 (100 to 700)
10 %	573	377 (351)	270 (100 to 700)
	2203	353 (344)	224 (100 to 700)
*p*-value		0.0075	

NOTE: *p*-values were obtained from the one-way analysis of variance, with three degress of freedom, conducted to compare the effect of risk level on willingness-to-pay

**Table 6:** Logistic regression: factors influencing the value of willingness-to-pay. Best fit logistic regression models for each cancer separately and all cancers together

£	All cancers together^a^			Colorectal (colonoscopy)			Lung (chest x-ray)			Pancreas (Ultrasound / CT scan)		
Variable (reference category)	Coeff (se)	*p*-value	95 % CI	Coeff (se)	*p*-value	95 % CI	Coeff (se)	*p*-value	95 % CI	Coeff (se)	*p*-value	95 % CI
Cancer (colorectal)		0.0012										
Lung	−50.80 (18.67)	0.0070	(−87.43 to −14.18)									
Pancreas	8.56 (19.37)	0.6590	(−29.44 to 46.55)									
Risk (1 %)		0.0206										
2 %	65.53 (21.07)	0.0020	(24.21 to 106.85)									
5 %	49.71 (21.29)	0.0200	(7.95 to 91.47)									
10 %	88.15 (20.93)	<0.0001	(47.10 to 129.20)									
Household Income (<£10,000)		<0.0001			<0.0001			<0.0001			<0.0001	
£10,000 - £25,000	46.96 (22.22)	0.0350	(3.39 to 90.53)	23.06 (27.35)	0.3990	(−30.58 to 76.71)	48.75 (26.63)	0.0670	(−3.48 to 100.99)	42.47 (27.29)	0.1200	(−11.05 to 96.00)
>£25,000	153.42 (25.43)	>0.0001	(103.55 to 203.30)	132.99 (28.73)	<0.0001	(76.62 to 189.36)	150.42 (29.83)	<0.0001	(91.90 to 208.93)	145.28 (28.86)	<0.0001	(88.66 to 201.90)
Education		<0.0001			<0.0001			0.0208			<0.0001	
GCSE or equivalent	−21.07 (23.59)	0.3720	(−67.33 to 25.20)	−9.85 (28.87)	0.7330	(−66.50 to 46.79)	−0.22 (27.73)	0.9940	(−54.61 to 54.17)	−41.75 (28.49)	0.1430	−97.64 to 14.14)
A-level or equivalent	21.48 (22.91)	0.3490	(−23.46 to 66.43)	33.44 (28.13)	0.2350	(−21.75 to 88.62)	35.68 (27.42)	0.1930	(−18.10 to 89.46)	−17.51 (28.02)	0.5320	−72.47 to 37.45)
Higher education	95.22 (24.36)	<0.0001	(47.44 to 143.01)	133.53 (29.96)	<0.0001	(74.74 to 192.31)	72.97 (29.08)	0.0120	(15.93 to 130.01)	86.06 (29.78)	0.0040	(27.64 to 144.49)
Cancer diagnosis (yes)		0.0033			0.0048			0.0138			0.0092	
No	−63.48 (21.67)	0.0030	(−105.98 to −20.98)	−73.04 (25.93)	0.0050	(−123.92 to −22.17)	−63.63 (25.92)	0.0140	(−114.47 to −12.79)	−70.71 (27.18)	0.0090	(−124.02 to −17.39)
Employment (Retired)		0.0137						0.0488				
Not in paid employment	−76.57 (27.55)	0.0060	(−130.60 to −22.53)				−82.45 (32.22)	0.0100	(−145.64 to −19.25)			
Working part time	−40.55 (21.33)	0.0570	(−82.37 to 1.27)				−43.00 (25.33)	0.0900	(−92.70 to 6.70)			
Working full time	−3.39 (21.22)	0.8730	(−45.01 to 38.23)				−20.24 (23.86)	0.3960	(−67.05 to 26.57)			
Family cancer (yes)								0.0276				
No							−46.89 (21.36)	0.0280	−88.78 to −5.00)			
Sex (male)											0.0298	
Female										−40.09 (18.49)	0.0300	−76.37 to −3.81)

^a^Based on first response only to avoid mote than one respinse from each participant

The results show that participants placed a lower value on an X-ray for lung cancer than the tests for colorectal or pancreatic cancer; the regression analysis in Table 6, which shows the best-fit logistic regression models, indicate that, controlling for other factors, there was a difference of about £51 (95 % CI: £14 to £87) in the mean willingness-to-pay between an X-ray and a colonoscopy and slightly less between an X-ray and the tests for pancreatic cancer. In general, testing was valued more highly when risk was high than when it was low, though the increase is not monotonic in the case of colorectal and pancreatic cancers (Tables 5 and 6). This may be related to the lack of a clear gradient for these two, as evidenced by testing for a difference in mean willingness-to-pay by risk level (*p*-values: colorectal 0.71; pancreas 0.91).

Around one fifth of respondents chose “I would not pay anything” when completing the payment scale exercise. Of these, one half said they did not believe they should pay for health care and one third said they could not afford to pay. Comparing the lowest risk level (1 %) with the highest (10 %), more individuals reported not being able to pay at the higher level (32 % vs 26 %) and more said they did not think they should pay at the lower level (59 % vs 52 %).

### Factors influencing responses to individual cancers

Previous analysis of responses to the ‘yes/no’ question of whether participants chose to be tested indicated that age was an important factor in all three cancer models [11]. Other variables affecting this decision were travel time to nearest hospital (colorectal and lung), whether a family member or close friend had previously been diagnosed with cancer (colorectal and lung) and income (colorectal and pancreas). The willingness-to-pay exercise indicated a rather different set of variables influencing the values placed on the tests: age did not appear as a factor in any of the three models and neither did travel time (Table 6). Three variables did however contribute to the willingness-to-pay values in all three tests: those with a higher income, particularly those in the highest bracket, were prepared to pay more for testing, as were the more highly educated, and a previous diagnosis of cancer increased the valued placed on a test by between £63 (95 % CI: £13 to £114) (lung) and £73 (95 % CI: £22 to £124) (colorectal). Additionally, the values placed on an X-ray for lung cancer were affected by a family member or close friend having been diagnosed with cancer and employment status. Males were prepared to pay more for testing for pancreatic cancer than were women.

### Test-retest

Analysis of the test-retest data suggested a good level of agreement in terms of the binary choice question: 47 (99 %) of the 48 who took part gave the same response as to whether they would choose to be tested. The level of agreement for both parts of the willingness-to-pay element was lower. Forty-three of the 48 respondents entered the willingness-to-pay exercise on both occasions and chose a payment scale. Of these, 25 (58 %) chose the same band on both occasions. Forty-two participants gave two valid willingness-to-pay values from the bidding process and 14 (33 %) gave the same exact value at re-test.

## Discussion

### Key findings

The aim of this willingness-to-pay exercise was to enhance the responses to a simple 'yes/no' question about testing for cancer, by indicating a strength of preference. We hypothesised that we might be able to identify a threshold level of risk below which patients would prefer to wait and see how symptoms develop before being referred for further investigation. Although the overwhelming majority of respondents opted for testing for the three cancers at all levels of risk included, the responses to the willingness-to-pay exercise did not augment the results of the binary question as anticipated; in fact the results suggest that participants treated the two parts of the survey rather differently. The 'yes/no' component of the survey indicated a risk gradient in the case of colorectal cancer, which was not seen in the willingness-to-pay values given to pay for a colonoscopy, but there was evidence that risk influenced willingness-to-pay values for testing for lung cancer. Furthermore, the participant characteristics that affected the decision to opt for a test were different than those affecting the willingness-to-pay values.

### Explanations for the findings

In general, the willingness-to-pay values obtained did not differentiate across risk levels as much as hypothesised. It is possible that the known “fear of cancer” may have contributed to this, indeed this was indicated in the yes/no part of the questionnaire where results suggested that patients would opt for testing even at very low levels of risk.

The willingness-to-pay values obtained suggest that risk is an important consideration when patients are deciding whether to accept the offer of a test for cancer of the lung but not colorectal or pancreas, a finding at odds with those of the binary 'yes/no' decision, where risk was only evident in colorectal cancer. Colorectal cancer involves the most invasive test, which may explain this finding, but it would seem that in the willingness-to-pay exercise participants may have discriminated according to neither the burden of the test nor the prognosis (pancreatic cancer having the worst likely outcome), but possibly their perception of the cost of the test. They valued a chest X-ray more highly if the risk of cancer was high (£365 at 10 % risk) and lower if the risk was low (£305 at 1 % risk) and there was a clear gradient (*p*-value 0.049). This was not the case with the more expensive tests where there was no evidence of an overall gradient despite the value placed on a CT scan for pancreatic cancer at the 10 % risk level being somewhat higher than the value at 1 %. The tests were described in detail in the vignettes so it is possible that many participants recognised that a chest X-ray is less costly than a colonoscopy and a CT scan. This confirms the belief that willingness-to-pay questions tend not to be sensitive to the size or scope of benefits [28] but is counter to the finding that people tend to state a similar amount for any reduction in risk of death or injury [29].

This raises the question of what people are actually valuing when they answer a willingness-to-pay question. Our hypothesis was that the willingness-to-pay exercise could be used to discriminate between cancers and risk levels more sensitively than a binary choice of whether to be tested or not. However, it would seem that participants viewed the two parts of the questionnaire separately and differently: the initial choice of whether to be tested appears to have been driven by the burden placed on them in undergoing the diagnostic test, illustrated by greater reservation about agreeing to a colonoscopy than the other tests, whereas the willingness-to-pay component appears to reflect people’s perception of the cost burden of the test, as a chest X-ray is by far the least expensive of the three. While the main aim of our study was to make comparisons across cancers and risk levels it is useful to reflect on the absolute levels of the willingness-to-pay values in comparison to those found in similar studies. Marshall et al. [5] compared physician and patient preferences for different methods of screening for colorectal cancer in Canada and the US. The values obtained ranged from US$111 (equivalent to £245 inflation adjusted) to C$232 (£662) depending on the type of test and in another US-based study to determine the value of time and discomfort of a colonoscopy, Jonas et al. [30] reported a mean value of US$263 (£563). In the UK Frew et al. [31] compared different methods of eliciting the willingness-to-pay for a faecal occult blood (FOB) test and a flexible sigmoidoscopy (FS) for colorectal cancer screening with results ranging from £86 (£136, inflation adjusted) for FS to £130 (£205) for FOB. In comparison to this, Nuemann et al. [17] obtained somewhat higher values in their US study for predictive testing: for breast and prostate cancer these ranged from US$508 (£904) for an imperfect test for breast cancer when the risk is 10 % to US$622 (£1007) for a perfect test for prostate cancer when the risk is 25 %. Our results of £305 to £393 across all cancers and risk levels fall towards the lower end of these, closer to the UK study results than those from North America.

### Methodological considerations

A variety of willingness-to-pay methods have been used in different settings and different patient groups [12]. In this study we used a bidding approach; furthermore, to mitigate the effect of any possible starting point bias we employed a two-part approach with each participant effectively selecting their own starting point. To our knowledge, this is a novel approach, made possible because of the mode of administration and the electronic nature of the questionnaire. However, in avoiding one methodological difficulty we have arguably introduced another. The bidding exercise was designed to restrict participants to the boundaries of the payment scale they chose in the first part of the willingness-to-pay exercise. This decision was largely driven by concern over ethics: it was considered ‘unfair’ to allow respondents to bid above/below the top/bottom value of the scale they had chosen. In fact, we found that 68 % of participants bid up to the highest value, leaving the unanswered question of what would have happened if they had been allowed to go beyond that value. This, in part, may explain the poor result in the relevant part of the test-retest exercise because once the participant had chosen a different payment scale in the retest, which 40 % did, it was impossible for them to identify the same final value as in the initial exercise.

In designing our study we were conscious of the phenomenon of ‘prominent’ numbers and we felt that the two-part design might mitigate this. In fact, analysing the results of the test-retest exercise we found that all participants who chose exactly the same value on both occasions had chosen a ‘prominent’ number: £0, £100, £300, £700 or £1000. This highlights the need for a better understanding of the role of ‘prominent’ numbers in such studies and has implications for the design of future studies.

We included reference goods in the payment scale exercise to help participants think about the value of a test. The verbal probing exercise did not throw up any consensus concerns about the use of reference goods or the choice of goods but there were some interesting individual comments: “*You are given a few ideas to give you the value of the cost”, “I think health is more important than material things”* and *“At my age we already have most things we need – furniture etc.”* However, when asked how easy or hard it was to put a value on the test none of the respondents mentioned that the reference goods helped. Whilst it seems useful to have benchmark reference goods for respondents to use our experiences suggest these are not essential; if they are included they must be chosen carefully and must be relevant to the population being surveyed.

### Limitations

The technique of willingness-to-pay is a conceptually attractive method of eliciting valuations and preferences. If responses reflect the true value that a population places on an intervention such as diagnostic testing the value can be compared with cost in a ‘purer’ way than any other outcome and used to make decisions about allocative efficiency. However, the results of this study show that many people find it difficult to think in terms of the value of benefit offered rather than what the intervention involves, and this is likely to be particularly true in a system of universal health insurance such as the NHS in England. While we were unable to use the results of this study as intended, they do reveal interesting unanswered questions which should be explored further. Qualitative methods could be employed to understand more about the thought processes and motivation of respondents as they complete such a survey; although some limited work has been done in this area [24, 32] there is a clear gap in our knowledge that needs to be closed in order to successfully exploit the full potential of willingness-to-pay as a technique for eliciting true valuations.

## Conclusion

The willingness-to-pay exercise reported here successfully obtained valuations for cancer testing from a large and diverse sample of the UK consulting population. A risk gradient was found only in the case of an X-ray for lung cancer, with higher values reflecting greater risk. This was inconsistent with responses to the question of whether to be tested or not, which suggested risk affected testing preferences only in the case of colorectal cancer. More investigation is needed to understand how patients perceive and respond to risk in this context, and how best to develop the use of willingness-to-pay techniques, which have the potential to provide good quality evidence which could enhance decision making in the provision of health care services.
